# Psychometric properties of a Swedish translation of the VISA-P outcome score for patellar tendinopathy

**DOI:** 10.1186/1471-2474-5-49

**Published:** 2004-12-18

**Authors:** Anna Frohm, Tönu Saartok, Gunnar Edman, Per Renström

**Affiliations:** 1 Elite Sports Centre, Swedish Sports Confederation, Bosön, Sweden; 2 Department of Surgical Sciences, Section of Sports Medicine, Karolinska Institute, SE-171 76 Stockholm, Sweden; 3 Department of Psychiatry, R & D Section, Danderyd Hospital, SE-182 87 Danderyd, Sweden

## Abstract

**Background:**

Self-administrated patient outcome scores are increasingly recommended for evaluation of primary outcome in clinical studies. The VISA-P score, developed at the Victorian Institute of Sport Assessment in Melbourne, Australia, is a questionnaire developed for patients with patellar tendinopathy and the patients assess severity of symptoms, function and ability to participate in sport. The aim of this study was to translate the questionnaire into Swedish and to study the reliability and validity of the translated questionnaire and resultant scores.

**Methods:**

The questionnaire was translated into Swedish according to internationally recommended guidelines for cross-cultural adaptation of self-report measures. The reliability and validity were tested in three different populations. The populations used were healthy students (n = 17), members of the Swedish male national basketball team (n = 17), considered as a population at risk, and a group of non-surgically treated patients (n = 17) with clinically diagnosed patellar tendinopathy. The questionnaire was completed by 51 subjects altogether.

**Results:**

The translated VISA-P questionnaire showed very good test-retest reliability (ICC = 0.97).

The mean (± SD) of the VISA-P score, at both the first and second test occasions was highest in the healthy student group 83 (± 13) and 81 (± 15), respectively. The score of the basketball players was 79 (± 24) and 80 (± 23), while the patient group scored significantly (p < 0.05) lower, 48 (± 20) and 52 (± 19).

**Conclusions:**

The translated version of the VISA-P questionnaire was linguistically and culturally equivalent to the original version. The translated score showed good reliability.

## Background

### Patellar tendinopathy

Patellar tendinopathy affects athletes in many sports and at all levels of participation, but is of particular concern for elite jumping athletes [1]. Many different types of sport activities have an increased risk for overuse of the patellar tendon including endurance sports (e.g. long-distance running and cross-country skiing) and sports with repetitive demands on strength and technique (e.g. tennis, baseball, volleyball, basketball and ballet) [2]. Athletes who participate in these sports may develop anterior knee pain that presents as tenderness at the inferior pole of the patella. This clinical syndrome is commonly called Jumper's knee, or patellar tendinopathy [3]. The term tendinopathy is considered to be the most appropriate clinical description for these chronic painful tendon conditions since there is no evidence of an inflammatory reaction in the chronically degenerated tendon [4,5]. The changes in the tendon are mainly due to chronic collagen fiber degeneration [6], but the cause and source of the pain still remains unclear.

There are few studies on non-surgical treatment of patellar tendinopathy and there is a lack of evidence-based knowledge evaluating the therapy [7]. In-vivo studies in human or animals indicate possible benefits from treatments like heavy pressure [8], therapeutic ultrasound [9] and eccentric strength training [10-14].

### Self-rated inventories for knee function

Patient-administrated questionnaires are frequently applied as primary outcome measures in clinical trials and several inventories have been translated from English into Swedish [15-17]. The WOMAC osteoarthritis index has been tested for reliability and validity in Sweden [18] and compared to quality of life instruments (SF-36 and NHP) [19]. The Knee injury and Osteoarthritis Outcome Score (KOOS) is also a self-administrated instrument measuring outcome after knee injury at impairment, disability, and handicap level with five subscales [16]. Garratt et al determined that KOOS showed good evidence of reliability, validity and responsiveness, and is recommended the score for knee diagnosis like ACL reconstruction, total knee replacements and for arthroplasty patients [20]. The only published clinical scale for patellar tendinopathy problems (VISA-P) was developed in Australia by the Victorian Institute of Sport Assessment in Melbourne [21]. The aim was to assess symptoms, simple tests of function and the ability of subjects to undertake sports. This self-administrated questionnaire has been documented as a reliable instrument for monitoring the progress of rehabilitation [3,7]. It has also been shown to be a valuable tool in the assessment and documentation of recovery from patellar tendinopathy [22]. Even so, the responsiveness and validity of the questionnaire have not yet been fully proven.

The purpose of this study was to translate and cross-culturally adapt the VISA-P score for a Swedish population and to perform a psychometric analysis as well as reliability and initial validity testing of the Swedish VISA-P score.

## Methods

### Subjects

Fifty-one subjects gave informed consent to participate in this study. The VISA-P score was administered to 17 healthy students [9 women, 8 men, mean age (± SD) 24 (± 6)]; a population at risk, the Swedish male national basketball team [17 men, mean age 26 (± 3)], and patients with the diagnosis patellar tendinopathy [17 men, mean age 22 (± 5)].

The study was approved by the Ethical Committee at the Medical Faculty of the Karolinska Institute, Stockholm (Dnr 00-103).

### The VISA-P score

The VISA-P score consists of eight questions [21], of which six questions concern pain experienced during a range of everyday activities. Two questions deal with the ability to engage in sport activities. All questions are answered on separate scales (0–10), where a higher score indicates a lower level of pain or impairment (Appendix A) [see additional file 1]. The maximal total score is 100 points, which would indicate that the person has no knee pain, good function and can perform fully in sports. The theoretical minimum score is 0 points.

The original VISA-P score lacks information about the selection of items, weighting of each answer and the ranking of the options in the subscales in question 8.

The aim of the present investigation was to get a "working tool" for further studies of the usefulness of the instrument for patellar tendinopathy patients in Sweden.

### Translation procedure

The VISA Tendon Study Group at the University of Melbourne in Australia was informed and gave their consent to a Swedish translation of their original VISA-P score (Karim Khan, personal communication, 2003).

The translation process followed the method described by Beaton et al [23]. This method is currently used by a number of organizations, including the American Association of Orthopaedic Surgeons (AAOS) Outcomes Committee as they coordinate translations of the different components of their outcome batteries [23]. The translation process is divided into five different stages: (I) Translation, (II) Synthesis, (III) Reverse translation, (IV) Expert committee review and (V) Pre-testing.

Initially, two physiotherapists performed two independent translations (I) from English into Swedish. A synthesis (II) of these translations was made, and the consensus of the two translated Swedish versions was documented. Reverse translations (III) were performed independently by three native Anglophones fluent in Swedish. One of the reverse translators was a physiotherapist, one was an economist and the third was a teacher. The three physiotherapists in the expert committee (IV) then made a semantic and idiomatic equivalence analysis between the original source and target Swedish version of the VISA-P questionnaire. The translated questionnaire was pre-tested (V) on 12 individuals, six patients with patellar tendinopathy and six physical education students.

### Test-retest reliability

The Swedish VISA-P score (Appendix B) [see additional file 2] was administrated to all 51 participants at Bosön, the Swedish National Sports Confederation Centre (Lidingö, Sweden). The participants completed the questionnaire twice within an interval of one week (range 4–7 days).

The principal investigator administrated the questionnaires at all test occasions, with the exception of six of the tendinopathy patients.

### Validity

For validity, the factor structure of the VISA-P score was analyzed with a principal component analysis, Varimax rotation. The number of extracted factors was equal to the number of eigen values above 1.00. Internal consistency of subscales, based on the factor analysis, and the total scale was calculated as a Cronbach α coefficient [24].

For discriminative validity of the VISA-P questionnaires were compared between three groups, each of which were expected to have different levels of scoring.

### Statistics

All variables were summarized according to standard descriptive methods [mean and standard deviation (SD)] and checked for outliers. No significant deviations from the normal distribution criterion were found. The test-retest reliability was analyzed according to the method described by Bland and Altman, which yields an intra-class correlation (ICC) [25]. Differences between test occasions and groups were analyzed with an ANOVA (analysis of variance for repeated measurements, group *time). In the post-hoc tests of group differences, Tukey's HSD method was applied. A significance level of five percent was applied (two-tailed).

## Results

### Translation

The expert committee considered the translation and reverse translation satisfactory.

### Test-retest reliability

**Figure 1:**
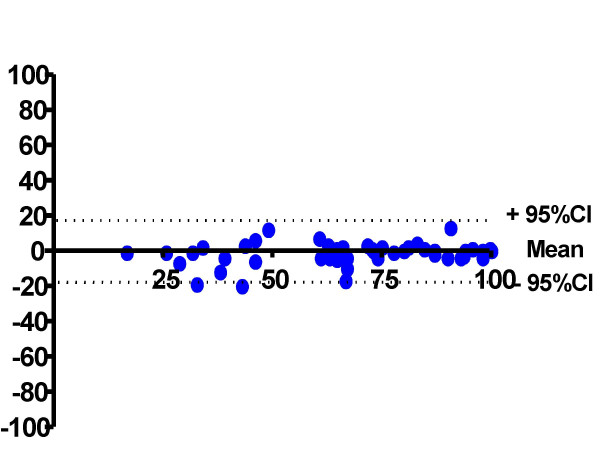
Bland-Altman plot of the Swedish VISA-P score in the reliability (test-retest within 1 week) investigation. Each blue dot indicates the difference in the total score, in relation to the mean score, between the two assessments.

The test-retest of the Swedish VISA-P score showed high reliability and significance (ICC = 0.97, p < 0.001). In Figure 1, the Bland-Altman plot is showing the difference in total score between occasion one (A) and occasion two (B), plotted against the mean value of both test occasions. There were no significant differences for the total VISA-P score between the first and second test occasions. Each question (Q) was analyzed separately regarding the reliability. Seven out of eight questions has a reliability of more than ICC = 0.8 (range 0.68–0.97).

The score was easy to use and it took about five minutes to complete.

### Internal consistency

The internal consistency of the total scale was high for the scores both at the first and second occasion, 0.83 and 0.82, respectively.

### Factor structure

The principal component analysis yielded a two-factor solution. The communality, i.e. the degree of explained variance, of one of the questions (Table 1, "sit pain-free?") was below 0.35, and thus not sufficiently explained by this solution. Thus, a three-factor solution was preferred which explained 85% of the total variance, with all communalities above 0.60. The first component comprised of six questions. The second and third components comprised of one question each. This solution showed high stability, being invariant in a second factor analysis of the scores from the second occasion (the amount of explained variance was 83%).

### Group differences in the VISA-P score

At the first test occasion (A) the mean (± SD) of the VISA-P score in the healthy student group was 83 (± 12), in the basketball players 79 (± 23), and 47 (± 20) in the patient group (Table 2). In all questions, the patient group had lower scores as compared to the other two groups and statistical significance (p < 0.05) was observed in all individual questions except the first ("sit pain-free"). In Table 1 the post-hoc tests for group differences are presented. The questions concerning pain ("pain during 10 single leg hops") had the greatest difference between the groups (F = 12.7, p < 0.001). Both activity questions ("currently undertaking sport" and" pain during activity") showed significant (p < 0.001) differences between the groups.

## Discussion

### Translation

The expert committee of the translation process expressed a general agreement of all the questions except one (Q1). During the translation procedure of the VISA-P score, the translation for "pain" was debated. Different Swedish words were discussed and compared between the different translators. Translations into the mother tongue, or the first language, more accurately reflected the nuances of the language. Reverse translation into English of the Swedish VISA-P version was without remarks. Thus, the original and translated versions were judged by the expert committee to be congruent.

### Test-retest reliability

Over a time interval of one week (range 4–7 days), the Swedish version of the VISA-P score showed high reliability (ICC = 0.97). As compared to other test-retest investigations of this score, this interval is the longest that has been studied [21].

### Validation of the VISA-P

A factor analysis yielded three factors, of which the first showed the highest correlations with two questions ("pain during a full weight bearing lunge" and "problems squatting", see Table 1). The two other factors comprised only one question each, "currently undertaking sport" and "sitting pain-free", respectively. The separate factor for the question about "sitting pain-free" may be an artefact, as this item was the first one where misperceptions of the response dimension were more likely, thereby increasing the risk of higher error or unique variance. Some subjects in the pre-testing group, reported that they had perceived high scores as more pain. Conceptually, this question is equivalent to the questions of the first component. Experiences from the pre-testing resulted in a more detailed instruction for filling out the Swedish questionnaire.

### Group difference

The patellar tendinopathy patients scored lower for all questions in the VISA-P score. The basketball players scored higher than the healthy students in two questions ("sitting pain-free" and "currently undertaking sport", see Table 2). The first question was the only question that did not show any statistical significance between the groups and, noteworthy, the lowest score, i.e. highest degree of problem. The reason given above regarding the risk of misperception of the response dimension might be an explanation.

The VISA-P score has not yet been validated for pathological knee conditions other than patellar tendinopathy. Considering the separate questions (Appendix A) [see additional file 1] it would be of interest to test the VISA-P score for patients with anterior knee pain other than patellar tendinopathy.

The significantly higher scores of the basketball players in question 7, "currently undertaking sport" (Table 2) were trivial and obvious, since all of them were active players in Swedish the national team. The standard deviation was nearly twice as high for the patients and basketball players as compared to the healthy students. This reflects the heterogeneity of the first two groups.

Generally, there is a debate concerning scores about the relevance of using the total score or dividing the score in different subgroups. A short clinical scale is often an advantage. The factor analysis as well as the analysis of differences between the groups suggests that the VISA-P score could be abbreviated to two or three items without losing significant clinical information (Table 1).

An important aspect of a clinical scale is its sensitivity for change or its ability to follow amelioration or exacerbation during treatment. The theoretical range of the VISA score, i.e. the floor and ceiling, is 0–100. The mean total score of the patients was approximately 50 (with a minimum value of 16) and for the control groups 80 (with a maximum value of 100. Thus, there seems to be sufficient scope to follow treatment effects, as well as to follow deteriorations of a risk group. It should be noted, however, that the present study was not designed to study treatment effects or development of a pathological process. The conclusion regarding the sensitivity of the VISA score, thus, awaits empirical support.

Although the mean VISA-P scoring was significantly different between asymptomatic subjects and patients with patellar tendinopathy, the score is not suggested to be a diagnostic test [21]. Therefore the score is considered to be suitable for group and intra-individual comparisons but should be avoided in inter-individual comparison. Another limitation of the score has not been shown to be applicable in a non-athletic population.

Adaptation of a questionnaire for use in a new setting is time consuming and costly. There are specific criteria that investigators should apply when evaluating patient-based outcome measures [26]. That being the case, larger international data collections and better correlations can be made when proper translations are performed and evaluations conducted. Additionally, there is a need for international accepted 'golden standards' in outcome scores.

In conclusion, the results of the present study suggest that the translated Swedish version (Appendix B) [see additional file 2] of the original Australian VISA-P score (Appendix A) [see additional file 1] had satisfactory test-retest reliability when used to evaluate symptoms, tests of function and ability to undertake sport in patients with patellar tendinopathy.

## Authors' contributions

AF initiated the study, led the translation process and conducted all test occasions. TS and PR helped with general analysis and writing the article. GE guided and helped the main author with the statistical analyses of the data collected. All four of the authors read and approved the article.

## Pre-publication history

The pre-publication history for this paper can be accessed here:



## Supplementary Material

Additional File 1 Appendix A. The original VISA-P score. Click here for file

Additional File 2 Appendix B. The translated and cross-culturally adapted Swedish VISA-P score. Click here for file

## Figures and Tables

**Table 1:** Three-factor solution according to a principal component analysis, Varimax rotation. The questions are ordered after their factor loading.

	*Component*			
		
*Question*	*I*	*II*	*III*	*h*^2^
Q4. Do you have pain when doing a full weight bearing lunge?	0.92	0.18	0.06	0.88
Q5. Do you have problems squatting?	0.90	-0.09	0.25	0.88
Q6. Do you have pain during or immediately after doing 10 single leg hops?	0.88	0.13	0.26	0.86
Q3. Do you have pain at the knee with full active non-weight bearing knee extension?	0.77	0.26	0.12	0.68
Q8. For how long can you manage being train/physically active?	0.75	0.39	0.11	0.73
Q2. Do you have pain walking downstairs with a normal gait cycle?	0.70	0.38	0.24	0.69
Q1. For how many minutes can you sit pain free?	0.23	0.13	0.96	0.99
Q7. Are you currently undertaking sport or other physical activity?	0.17	0.95	0.11	0.94

**Table 2:** Mean score and standard deviation (SD) in three groups of subjects: patients with patellar tendinopathy (P); basket-ball players (B); and healthy students (H), and analysis of variance (ANOVA) of group differences of the Swedish VISA score.

		*Patients (n = 17)*		*Basketball (n = 17)*		*Healthy student (n = 17)*		*Total (n = 51)*		*ANOVA*		
					
*Question*	*Occasion*	*M*	*SD*	*M*	*SD*	*M*	*SD*	*M*	*SD*	*F*	*p*	*Tukey*
Q1. For how many minutes can you sit pain free?	A	5.41	2.94	7.53	2.88	7.24	3.52	6.73	3.21	2.29	n.s	ns
	B	6.06	2.80	7.82	2.65	7.18	3.63	7.02	3.08			
Q2. Do you have pain walking down-stairs with a normal gait cycle?	A	5.94	2.35	8.47	2.81	8.94	1.34	7.78	2.58	8.70	p < 0.001	P < B;P < H
	B	6.94	2.02	8.82	1.98	8.41	2.18	8.06	2.18			
Q3. Do you have pain at the knee with full active non-weight bearing knee extension?	A	6.59	3.00	8.47	2.53	9.53	1.06	8.20	2.61	6.84	p < 0.05	P < H
	B	6.29	2.44	8.82	1.97	9.18	1.33	8.10	2.33			
Q4. Do you have pain when doing a full weight bearing lunge?	A	4.41	2.89	7.65	3.08	8.06	2.19	6.71	3.16	8.97	p < 0.05	p < B;p < H
	B	5.24	2.86	7.88	2.83	8.00	2.24	7.04	2.91			
Q5. Do you have problems squatting?	A	5.76	2.28	7.35	3.26	8.24	1.98	7.12	2.71	4.05	p < 0.05	p < H
	B	6.65	2.47	7.76	2.88	8.18	2.27	7.53	2.58			
Q6. Do you have pain during or immediately after doing 10 single leg hops?	A	3.29	2.33	6.65	3.62	8.18	2.55	6.04	3.50	12.66	p < 0.001	p < B;p < H
	B	4.29	2.87	6.71	3.88	8.18	2.04	6.39	3.38			
Q7. Are you currently undertaking sport or other physical activity?	A	5.00	2.48	9.47	1.58	7.35	2.98	7.27	3.00	14.57	p < 0.001	p < H<B
	B	4.41	2.62	9.47	1.58	7.00	2.81	6.96	3.14			
Q8. For how long can you manage being train/physically active?	A	11.35	10.94	23.41	8.27	25.53	5.68	20.10	10.50	13.54	p < 0.001	p < B;p < H
	B	12.41	10.25	22.82	8.13	25.53	5.68	20.25	9.86			

Total score	A	47.76	20.26	79.00	24.18	83.06	12.60	69.94	24.96	16.48	p < 0.001	p < B;p < H
	B	52.29	18.98	80.12	22.82	81.65	15.48	71.35	23.33			
