# Supplementary material for: Psychometric properties of a Swedish translation of the VISA-P outcome score for patellar tendinopathy
Source: BMC Musculoskelet Disord. 2004 Dec 18;5:49. doi: 10.1186/1471-2474-5-49 (PMC545958; doi:10.1186/1471-2474-5-49)
Supplement: Additional File 1 — Appendix A. The original VISA-P score. [file 1471-2474-5-49-S1.doc]

Name:

Age:

Date:

# Victorian Institute of Sport Assessement Scale

1. **For how many minutes can you sit pain-free?**

## Points

0 mins 100 mins

0 1 2 3 4 5 6 7 8 9 10

1. **Do you have pain walking downstairs with a normal gait cycle?**

## Points

Strong severe No pain

pain 0 1 2 3 4 5 6 7 8 9 10

1. **Do you have pain at the knee with full active non-weight bearing knee extension?**

## Points

Strong severe No pain

pain 0 1 2 3 4 5 6 7 8 9 10

1. **Do you have pain when doing a full weight-bearing lunge?**

## Points

Strong severe No pain

pain 0 1 2 3 4 5 6 7 8 9 10

1. **Do you have problems squatting?**

## Points

Unable No problem

0 1 2 3 4 5 6 7 8 9 10

**6.** **Do you have pain during or immediately after doing 10 single leg hops?**

## Points

Strong severe No pain

pain/ 0 1 2 3 4 5 6 7 8 9 10

unable

**7. Are you currently undertaking sport or other physical activity?** Points

0 Not at all

4 Modified training ± modified competition

7 Full training ­­­± competition but not at the same level as when symptoms began

1. Competing at the same or higher level when symptoms began

**8. Please complete EITHER A, B or C in this question.**

- If you have **no pain** while undertaking sport please complete **Q8a only**
- If you have **pain while undertaking sport but it does not stop you** from completing the activity, please complete **Q8b only.**
- If you **have pain that stops you from completing sporting activities,** please complete Q**8 c only**

**8a.** If you have no pain while undertaking sport, for how long can you train/practise?

NIL 0-5 mins 5-10 mins 11-15 mins >15 mins Points

**0 7 14 21 30**

**or**

**8b.** If you have some pain while undertaking sport, but it does not stop you from completing your training/practice, for how long can you train/practise?

NIL 0-5 mins 5-10 mins 11-15 mins >15 mins Points

**0 4 10 14 20**

**or**

**8c.** If you have pain that stops you from completing your training/practice, for how long can you train/practise?

NIL 0-5 mins 5-10 mins 11-15 mins >15 mins Points

**0 2 5 7 10**

**TOTAL VISA SCORE**
