# Supplementary material for: Psychometric properties of a Swedish translation of the VISA-P outcome score for patellar tendinopathy
Source: BMC Musculoskelet Disord. 2004 Dec 18;5:49. doi: 10.1186/1471-2474-5-49 (PMC545958; doi:10.1186/1471-2474-5-49)
Supplement: Additional File 2 — Appendix B. The translated and cross-culturally adapted Swedish VISA-P score. [file 1471-2474-5-49-S2.doc]

Namn:

Ålder:

Datum:

**Läs varje fråga noga två gånger och kryssa för det alternativ som passar Dig bäst!**

**Mycket smärta ger 0 poäng (vä), ingen smärta ger 10 poäng (hö).**

# Victorian Institute of Sport Assessement Scale

1. **Under hur många minuter kan du sitta SMÄRTFRITT ?**

## Poäng

0 minuter 100 minuter

0 1 2 3 4 5 6 7 8 9 10

1. **Har Du smärta vid gång nedför i trappor?**

## Poäng

Mycket Ingen smärta

smärta 0 1 2 3 4 5 6 7 8 9 10

1. **Har Du smärta vid en icke belastad knästräckning?**

## Poäng

Mycket Ingen smärta

Smärta 0 1 2 3 4 5 6 7 8 9 10

1. **Har Du smärta vid ett utfallssteg?**

## Poäng

Mycket Ingen smärta

smärta 0 1 2 3 4 5 6 7 8 9 10

1. **Har Du smärta vid tvåbens knäböj ?**

## Poäng

Går ej Inga problem

0 1 2 3 4 5 6 7 8 9 10

**6.** **Har Du smärta under eller direkt efter 10 enbenshopp?**

## Poäng

Går ej Ingen smärta

p.g.a 0 1 2 3 4 5 6 7 8 9 10

smärta

**7. Utövar du för närvarande någon idrott eller annan fysisk aktivitet?** Poäng

0 Ingen träning/ fysisk aktivitet

4 Begränsad träning ­­­ ± begränsad tävling

7 Full träning ­­­ ± tävlar men ej på samma nivå som när symtomen började.

1. Tävlar på samma eller högre nivå som när symtomen började.

**8. Vänligen svara antingen på A, B eller C i den här frågan**

- Har Du **ingen smärta** under idrott svara **endast** på fråga **8a.**
- Har Du **smärta** under idrott men det **inte hindrar Dig från att vara aktiv** svara **endast** på fråga **8b.**
- Har Du **smärta** som **hindrar Dig från att idrotta**, svara **endast** på fråga **8 c.**

**8a.** Om Du **inte har någon smärta** under idrott, hur länge kan du träna/vara fysiskt aktiv?

Ingen 0-5 min. 5-10 min. 11-15 min. >15 min. Poäng

**0 7 14 21 30**

**eller**

**8b.** Om Du har **lite** **smärta under aktivitet**, men det **inte hindrar Dig** från din träning, hur länge kan

Du träna/vara fysiskt aktiv?

Ingen 0-5 min. 5-10 min. 11-15 min. >15 min. Poäng

**0 4 10 14 20**

**eller**

**8c.** Om **Du har smärta** som **hindrar Dig** från att fullfölja din träning, hur länge kan Du träna

/vara fysiskt aktiv?

Ingen 0-5 min. 5-10 min. 11-15 min. >15 min. Poäng

**0 2 5 7 10**

**TOTAL VISA SCORE**
